# Relationship Between Iron Deficiency Anemia and Stunting in Pediatric Populations in Developing Countries: A Systematic Review and Meta-Analysis

**DOI:** 10.3390/children11101268

**Published:** 2024-10-19

**Authors:** Caroline Oktarina, Charisma Dilantika, Nova Lidia Sitorus, Ray Wagiu Basrowi

**Affiliations:** 1 Faculty of Medicine, Universitas Indonesia, Depok 16424, Jawa Barat, Indonesia; caroline.oktarina91@ui.ac.id; 2 Danone Specialized Nutrition Indonesia, South Jakarta 12930, Indonesia; charisma.dilantika@danone.com (C.D.); nova.sitorus@danone.com (N.L.S.); 3 Department of Community Medicine, Faculty of Medicine, Universitas Indonesia, Depok 16424, Jawa Barat, Indonesia

**Keywords:** iron deficiency anemia, stunting, children, risk factors, relationship

## Abstract

Background/Objectives: Iron deficiency anemia (IDA) and stunting are prevalent global health issues, particularly in developing countries, where previous studies have suggested a potential relationship between them. This systematic review aims to analyze the relationship between iron deficiency anemia and stunting in pediatric populations in developing countries. Methods: Literature searches were conducted on PubMed, EMBASE, Cochrane Library, and EBSCO Host. The primary outcome was the association between IDA and stunting. Risk of bias was assessed using the Newcastle–Ottawa Scale (NOS) for cohort studies and the Effective Public Health Practice Project (EPHPP) for other observational studies. Meta-analysis was performed with a random-effects model and heterogeneity assessment. A Grading of Recommendations, Assessment, Development, and Evaluation (GRADE) assessment was performed to determine the certainty and importance of the study. Results: Out of 19,095 articles, 15 studies were included in the systematic review, and 4 studies were included in the meta-analysis, encompassing 21,936 subjects aged 0 to 12 years. IDA prevalence ranged from 3.6 to 58.8%, while stunting prevalence varied from 6.6 to 44.5%. Nine articles supported a significant relationship between IDA and stunting, revealing that stunted children had a 1.31–6.785 times higher risk of developing IDA. The odds ratio of children with IDA to be stunted was 2.27 (95% CI = 1.30–3.95). All studies exhibited a moderate risk of bias. GRADE assessment suggested that the evidence’s certainty is low but important. Conclusions: The high IDA prevalence in developing countries, including Indonesia, is associated with stunting in children, suggesting a synergistic relationship.

## 1. Introduction

Iron deficiency anemia (IDA) arises from insufficient iron intake, leading to a decrease in hemoglobin concentration exceeding two standard deviations below the established average population value. It is a global health concern, particularly affecting children. It is associated with significant morbidity in children, namely growth and development disorders [1,2]. The World Health Organization (WHO) estimates that a quarter of the global population suffers from anemia, with half of these cases attributed to iron deficiency and with a higher prevalence in developing countries due to limited resources [3]. Cambridge Dictionary defines a developing country as “a country with little industrial and economic activity and where people generally have low incomes [4]”. In children younger than five years old, the global estimated prevalence of IDA is 39.8%, while the prevalence is higher in South Asia (52%) [5]. Indonesia is a lower–middle income country residing in Southeast Asia. There are limited studies on the prevalence or incidence of IDA in Indonesia. A previous study in Indonesia showed that the prevalence of IDA was 29.4%, 16%, and 15.2% in children aged 6–59 months old, 5–11.9 years old, and 12–19 years old, respectively [6]. Another study in children aged 6–18 years old living in low socioeconomic settings showed that the prevalence of IDA was 5.8% [7].

Stunting, impaired growth manifested by low height for their age in children, is also a significant global health problem [8,9]. Although global stunting prevalence in children under five has decreased from 39.3% in 1990 to 21.3% in 2019, South Asia and Sub-Saharan African countries still contribute to this figure significantly [10]. Indonesia reported stunting cases in 37% of the population of children in 2013. However, the prevalence varied between provinces, which was 26% in Riau and 52% in East Nusa Tenggara [11].

Both IDA and stunting are critical health issues [3,11], particularly in developing countries like Indonesia, where more than a quarter of the pediatric population is affected [7,11]. Previous studies have highlighted the association between IDA and stunting, with stunting considered a risk factor for developing IDA [12,13]. However, there are inconsistent results across studies. Most studies were conducted with a cross-sectional design, which cannot produce evidence of causality. In addition, some studies have small sample sizes [12,13]. To date, there has been no meta-analysis on the relationship between iron deficiency anemia and stunting, particularly in pediatric populations. The current systematic review and meta-analysis aim to analyze the relationship between iron deficiency anemia and stunting in pediatric populations in developing countries.

## 2. Materials and Methods

### 2.1. Search Strategy

This study adheres to the Preferred Reporting Items for Systematic Reviews and Meta-analysis (PRISMA) 2020 guidelines. A literature search was conducted in November 2023 on PubMed, EMBASE, Cochrane Library, and EBSCO Host using specific search terms. The search terms are “(((iron deficiency anemia) AND (stunting)) AND (children)) OR (pediatric)) AND (developing countries)”. These search terms correspond to the following Population (P), Intervention (I), Comparison (C), and Outcomes (O):

P: Children in developing countries;

I: Iron deficiency anemia;

C: No iron deficiency anemia;

O: Stunting.

### 2.2. Inclusion and Exclusion Criteria

The inclusion criteria were studies on pediatric patients with IDA reporting its association with stunting in developing countries in the forms of observational cross-sectional studies, case–control studies, and cohort studies. The exclusion criteria were patients with comorbidities that could influence their nutritional status, such as cancer, cardiovascular diseases, and gastrointestinal diseases. Narrative reviews, case reports, opinions, letters to editors, proceedings, systematic reviews, meta-analyses, and gray literature were excluded from the study.

### 2.3. Data Extraction

Two investigators performed data extraction according to the PRISMA guidelines. The data included year of publication, sample size, sociodemographic characteristics of the subjects, nutritional status of the subjects, prevalence or incidence of IDA, prevalence or incidence of stunting, and statistics of association between IDA and stunting. The IDA should be established through laboratory examination, and stunting should be diagnosed according to the World Health Organization (WHO) or Center for Disease Prevention and Control (CDC) criteria. Disagreements between investigators regarding the inconsistent results were resolved by a third reviewer.

### 2.4. Selection of Studies and Eligibility Assessment

Screening of titles and abstracts was carried out independently by two reviewers with Rayyan QCRI. Another reviewer was tasked to supervise the screening process. The full texts of the selected articles were sought, and both reviewers performed eligibility assessments independently. There was no blinding on the study’s bibliographic information.

### 2.5. Risk of Bias

Numerous tools were employed to measure the risk of bias according to the type of the study. Two reviewers performed a risk of bias assessment and measured the evidence level of all studies. Disagreements between reviewers were resolved by a third reviewer. The Newcastle–Ottawa Scale (NOS) was used for the cohort study, while the Effective Public Health Practice Project (EPHPP) was used for other observational studies. The NOS assessed three domains, namely selection (maximum 4 points), comparability (maximum 2 points), and outcome (maximum 3 points). The EPHPP assessed seven domains, including selection bias (2 items), study designs (4 items), confounders (2 items), blinding (2 items), data collection (2 items), withdrawal/dropout (2 items), and analysis (4 items).

### 2.6. Data Analysis

Data analysis was performed by using the meta (version 6.5-0) and metafor (version 4.4-0) packages of RStudio software version 4.3.2 (RStudio, Inc., Boston, MA, USA). A random-effects model was utilized to calculate the odds ratio with a 95% Confidence Interval (95%CI). Heterogeneity among studies was assessed with I^2^ statistics with a result >75% representing substantial heterogeneity. A funnel plot was developed to identify publication bias.

### 2.7. Evaluation of Certainty and Importance of the Studies

This study utilized GRADEpro to determine the certainty and importance of the studies included. The grading assessed the risk of bias, inconsistency, indirectness, and imprecision. The studies included were assessed as very low, low, or moderate certainty and not important, important, or critical [14].

### 2.8. Ethical Approval

Ethical approval was unnecessary as this study did not involve animal or human research. This meta-analysis was registered in PROSPERO (registration number CRD42024557004).

## 3. Results

### 3.1. Study Flow

**Figure 1:**
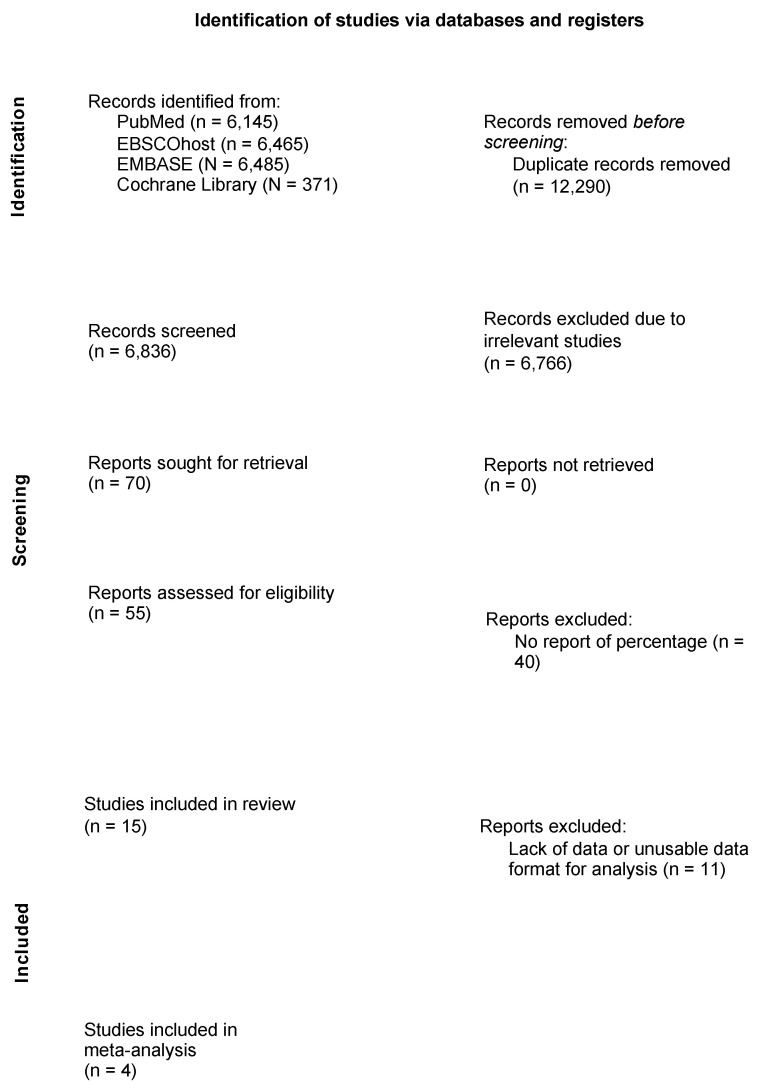
Literature search strategy based on the Preferred Reporting Items for Systematic Reviews and Meta-analysis (PRISMA) 2020.

Figure 1 describes the study flow diagram. A total of 19,095 articles were narrowed down to 6465 after removing duplicates. The full texts of seventy studies were retrieved and assessed for eligibility. Fifteen studies were included in this systematic review and four studies were included in the meta-analysis.

### 3.2. Characteristics of the Study

The studies, detailed in Table 1, included original articles, with the majority being cross-sectional. Only one study was retrospective. Geographically, seven studies were from Asia, seven from Africa, and one from South America. The total population comprised 21,936 subjects aged 0 to 12 years.

### 3.3. Prevalence of IDA

The IDA prevalence differs between studies. Studies in Asia reported varied results, ranging from 3.6% in Taiwan to 52.7% in Bangladesh, while African studies reported rates from 3.8% in South Africa to 58.8% in Tanzania. A study in Brazil reported a prevalence of 10.3%.

### 3.4. Prevalence of Stunting

Similar to IDA, the stunting prevalence differs between studies. Not all studies reported the stunting prevalence. Studies in Asia reported varied results, ranging from 39.8% in India to 44.5% in Pakistan. Studies in Africa reported rather better results, ranging from 6.6% in South Africa to 37% in Ethiopia. A study in Brazil reported a prevalence of 7.1% in children aged < 5 years and 3.7% in children aged ≥ 5 years old.

### 3.5. Relationship Between IDA and Stunting

**Figure 2:**
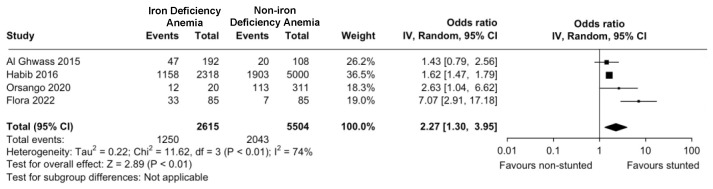
The incidence of stunting in children with iron deficiency anemia in developing countries [13,18,20,24].

**Figure 3:**
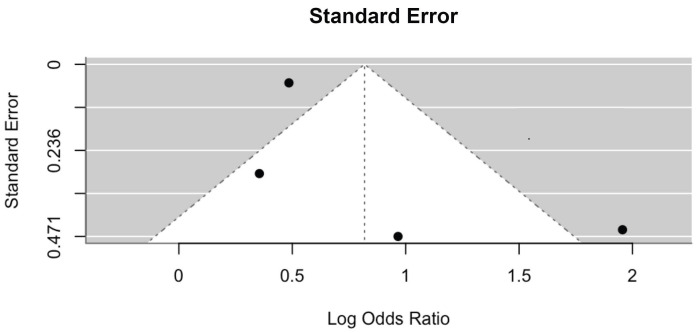
Funnel plot showing no evidence of publication bias.

Of the fifteen studies included, nine articles supported the significant relationship between IDA and stunting. Of six studies that did not support the relationship, only two studies assessed the direct relationship between IDA and stunting, while the other four studies assessed the relationship between IDA and height to age Z (HAZ) scores. From the systematic review, it is found that children with stunting had a 1.31–6.785 times higher risk of developing IDA. The meta-analysis showed that the odds ratio of children with IDA to be stunted was 2.27 (95% CI = 1.30–3.95) (Figure 2). Based on the forest plot, the four included studies showed no substantial heterogeneity (I^2^ = 74%), with no evidence of publication bias (Figure 3).

### 3.6. Comparison of Children Aged Younger than 60 Months Old and Older than 60 Months Old

Among the nine studies addressing the relationship between IDA and stunting, only one included children aged 9–12 years, showing a 6.785 times higher IDA risk in stunted children. Studies in children under 60 months old showed lower risks, ranging from 1.31 to 2.6 times.

### 3.7. Study Quality

**Figure 4:**
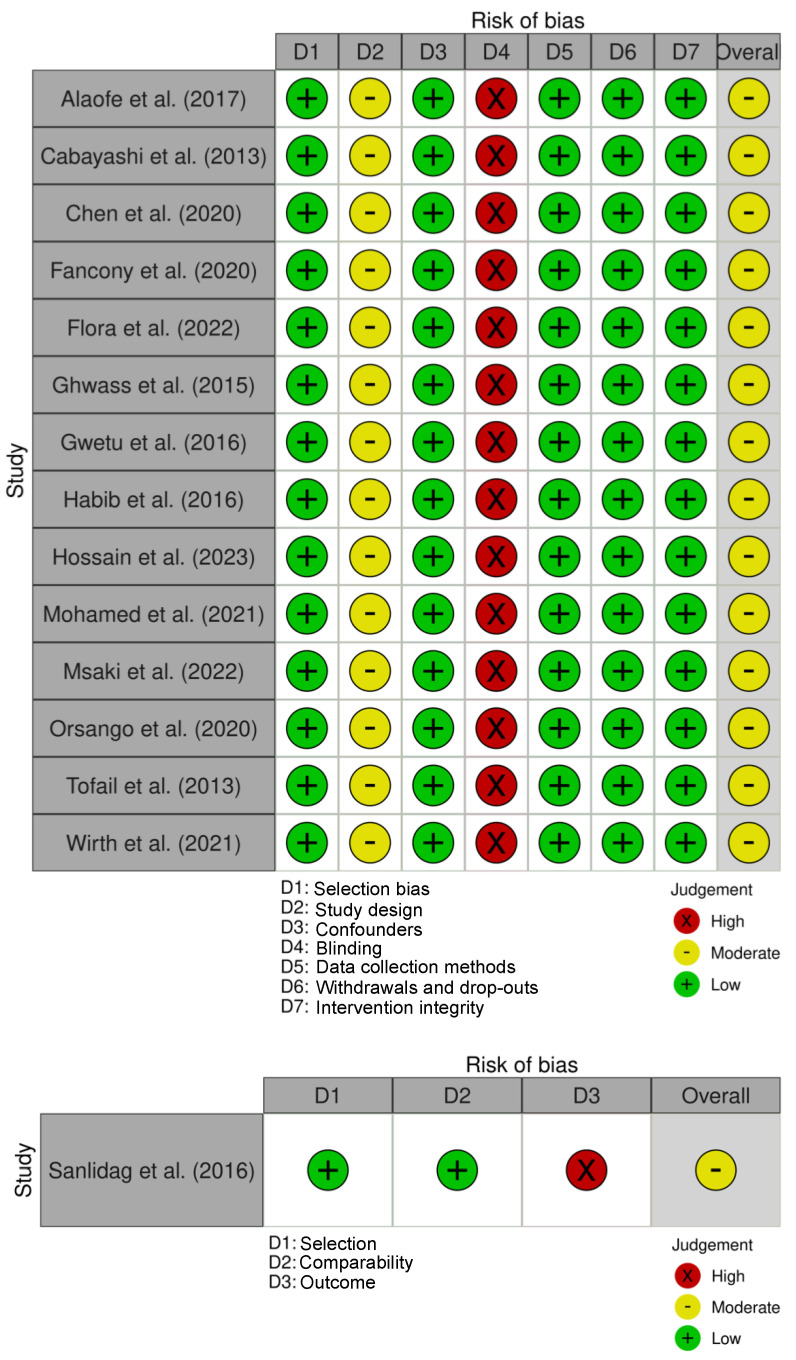
Risk of bias assessments of the studies [12,13,15,16,17,18,19,20,21,22,23,24,25,26,27].

The risk of bias assessment using EPHPP indicated overall moderate results. All 14 studies assessed with EPHPP were judged moderate in domain 2 (study design) due to their cross-sectional nature and high in domain 4 (blinding) due to a lack of blinding during the study. One retrospective study had a potentially high risk of bias in domain 3 (outcome) due to inadequate follow-up and no independent blind assessment. The results of the risk of bias assessment are shown in Figure 4.

### 3.8. Grading of Recommendations, Assessment, Development and Evaluation (GRADE)

The results of the GRADE assessment are shown in Table 2. Only three studies provided data on several patients with stunting in both IDA and non-IDA populations. Due to the limited data, the results should be interpreted cautiously. The assessment suggested that the evidence’s certainty is low but important.

## 4. Discussion

### 4.1. Epidemiology

This review shows that the prevalence of IDA in developing countries varies from 3.6% to 58.8%, with a higher prevalence in African countries [16,23]. The global estimate for IDA prevalence in preschool children is 23.5%. The prevalence is significantly higher in developing countries, especially in low-income ones [1]. A study from Jakarta, Indonesia, showed that IDA prevalence in children aged 6–18 years old was 5.8% [7]. Another study in Kalimantan, Indonesia, reported a higher prevalence: 29.4% in children aged 6–59 months old, 16% in those aged 5–11.9 years old, and 15.2% in those aged 12–19 years old [6]. A study from Somalia, one of the ten poorest countries in the world, reported a similar result, with an IDA prevalence of 28.6% in children aged 6–59 months old [27]. The epidemiology of infants aged younger than 6 months old is seldom reported because this population suffers from physiologic anemia. Older infants and children start to receive food which can alter their iron status if the diet is not proper [6].

### 4.2. Risk Factors of IDA

IDA is often identified in developing countries due to low socioeconomic status and poor resource settings. Dietary intake is the major source of iron; hence, an inadequate diet will lead to iron deficiency [13]. Apart from stunting, several risk factors are proven to be associated with IDA, including the age of 6–23 months old, the male gender, and the presence of inflammation. Children aged 6–23 months old need a higher iron intake. In this age group, children start to eat food to complement breastfeeding. It is important that at this age, the nutrients they obtain from the food should be adequate because breast milk itself might not be sufficient [12,16,18,28]. This becomes a problem, particularly in developing countries where adequate nutrients might not be available for a population with low socioeconomic status [18,20,25]. Regarding gender, it is thought that boys might have lower iron stores [17]. Inflammation might influence IDA through indirect associations. When chronic inflammation occurs, the erythropoiesis is suppressed, along with a shorter erythrocyte lifespan and elevated hepcidin levels. These processes hinder iron absorption. Special consideration should be taken when assessing IDA in children suffering from inflammation because serum ferritin acts as an acute-phase protein that is elevated during inflammation [27].

Indonesia is one of the developing countries with a significant number of pediatric IDA cases. A previous study in Indonesia reported that the average iron intake in children aged 6–23 months old was 4.6 mg, lower than standard nutritional requirement. Low iron intake is associated with low socioeconomic status, older age, living in a village, and breastfeeding weaning [29]. In addition, several factors influencing the high number of IDA cases in Indonesia include the following [30,31,32,33,34]:

1. Challenges in the implementation of iron supplementation for pregnant women.
2. The low iron content in breast milk.
3. The high prevalence of helminth infection in Indonesia, a tropical country with low socioeconomic settings.
4. Poor maternal education on nutrition and pregnancy-related healthcare services.
5. A lack of micronutrients and insufficient consumption of iron-rich foods. A fortification program has been developed for foods, particularly wheat flour and rice. However, the availability of fortified wheat flour is limited in certain regions of Indonesia.
6. No established guidelines or regulations for routine screening of iron status [30,31,32,33,34], especially in children.

Vitamin A deficiency is also linked to IDA. Both entities might coexist due to poor nutritional intake. However, vitamin A deficiency can further decrease the erythropoiesis process while the iron is also trapped in the liver, leading to IDA [27]. Another study also denotes the role of dental caries in inducing IDA in children because the pain induced by mastication will reduce food intake and lower their appetite. Red meat, which is a major source of iron, will be more difficult to consume, hence decreasing iron intake [22]. In addition, if the mother also suffers from iron deficiency during pregnancy, it is very likely that the children will be born with iron deficiency as well. A high prevalence of IDA is found in the children of mothers with low educational levels [18].

### 4.3. Relationship Between Stunting and IDA

**Figure 5:**
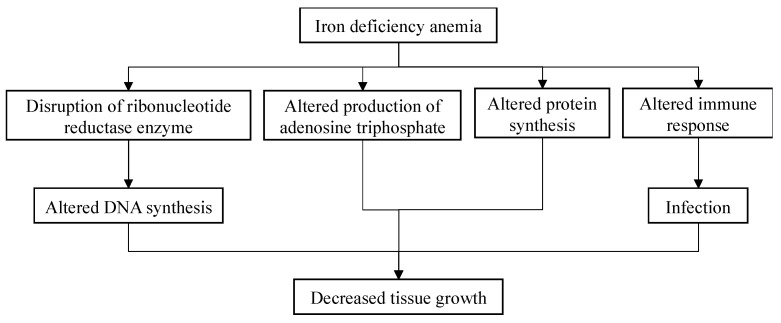
Potential mechanism of iron deficiency anemia influencing stunting [13].

Nine of the fifteen included studies supported the relationship between stunting and IDA [12,13,17,18,20,23,24,26,27]. The results of the meta-analysis support the hypothesis that children with IDA are more prone to being stunted compared to children without IDA, with a 2.27 times higher risk. A study by Al Ghwass et al. showed that 24.5% of the children with IDA were stunted, despite not finding a significant association [18]. While the published studies only showed how stunting increases the risk of IDA, IDA can also induce stunting. The suggested mechanism of how IDA influences children’s growth is depicted in Figure 5. Iron is a micronutrient which plays a role in tissue growth. As iron contributes to the synthesis of deoxyribonucleic acid (DNA), iron-deficient states will hinder tissue growth, leading to growth disruption. Another mechanism is that iron-deficient states make the body more prone to infection due to compromised immune responses. If the infection persists longer, it will also lead to growth disruption [13].

There were potential confounders that might contribute to stunting in the populations. As most studies were cross-sectional studies, chronic anemia or low iron intake might influence the results [19]. The small sample size in some studies might overestimate the association found [13,22,25]. An assessment of any other potential cause of stunting should also have been performed [18]. These potential confounders should be addressed for future studies.

### 4.4. Comparison of Stunting-Related IDA in Children Aged Younger than 60 Months Old and Older than 60 Months Old

An age younger than 60 months old is considered preschool age, in which the prevalence of IDA is high [1]. The IDA prevalence decreases in older age groups [6]. As children age, iron intake will increase due to the introduction of various foods containing iron, and immunity ensues so that the incidence of infection is less than in children of a younger age [35].

### 4.5. Screening and Examination for IDA

The diagnosis of IDA is defined as the presence of iron deficiency and anemia. Anemia is defined as a hemoglobin level of <11 g/dL. Iron deficiency is defined as a ferritin level <12 μg/L in children aged <5 years old or <15 μg/L in children aged ≥ 5 years old or serum transferrin > 8.3 mg/L [15,24]. Dietary assessment with a 7-day structured food frequency questionnaire and 24 h dietary recall questionnaire might be used as a screening tool for iron deficiency [24].

### 4.6. Complications and Long-Term Effects

Stunting and IDA are linked to problems in children’s development. Long-term associations of stunting at a younger age include cognitive disorder, difficulty in performing fine motor skills, disruption of psychomotor skills, and influence on neurosensory integration [13,26]. Stunting will affect the nerve cell’s development, which leads to development disruption [36].

Long-term associations of IDA were shown to induce reduced cognitive performance, motor deficit, and problems with cooperation. Iron treatment and stimulation did not significantly improve the conditions [21,26,37]. IDA is associated with dental caries because iron plays a role in oral defense mechanisms [22]. IDA is also proven to be associated with a lower intelligence quotient (IQ). A study in children aged under 5 years old showed that the percentage of children with below-average IQ was significantly higher in the IDA group compared to the control group (72% vs. 20%; *p* < 0.01). The study also denoted that two subjects in the IDA group had an intellectual disability (8%) [38]. Another study in older children aged 12–15 years old also showed that IDA subjects had significantly lower math scores (42.6 vs. 47.2; *p* = 0.02), knowledge (79.4 vs. 88; *p* = 0.001), working memory (92.1 vs. 96.7; *p* = 0.01), and full-scale IQ (89.9 vs. 93; *p* = 0.01) compared to normal subjects [39]. IDA is also associated with attention deficit hyperactivity disorder (ADHD). A study in children aged 5–18 years old showed that iron deficiency was a significant risk factor for ADHD (OR = 2.81; 95% CI = 1.72–4.53; *p* < 0.01) [40]. Iron and other micronutrients are essential for brain development, particularly in the first five years of life. Therefore, long-term iron deficiency can induce developmental disorders, similar to stunting [36]. Table 1 shows the long-term associations between IDA and stunting pediatric populations.

### 4.7. Limitations

The limitations of this systematic review and meta-analysis include that almost all studies had a cross-sectional design; additionally, not all studies employed the same method in assessing IDA and stunting. While a cross-sectional study can establish associations between variables, it fails to explain how the dependent variable is affected by the independent variable. Moreover, different assessment methods might influence the results. The assessment showed no publication bias; however, a small sample size should be taken into account for this result.

## 5. Conclusions

The prevalence of IDA is high in developing countries, including Indonesia. IDA is associated with stunting in children, which might be explained by the synergistic associations of IDA and stunting, where one can potentially induce the other. However, the majority of studies are cross-sectional. Therefore, further studies with a prospective design are necessary to establish the mechanisms by which IDA induces stunting and vice versa.

## Figures and Tables

**Table 1:** Summary of the included studies.

Author	Study Type	Country	Number of Participants	Sample Characteristics	IDA Assessment	Stunting Assessment	Outcomes
Alaofe et al. (2017) [12]	Cross-sectional study	India	647	Children aged 6–59 months old	Serum ferritin < 12 mg/L in reference group, <15 mL/L in incubation group and early convalescence group, or <22 mg/L in late convalescence group with hemoglobin < 11 mg/dL	WHO Growth Standards HAZ < −2.0 SD	21.2% of the subjects had IDA. The prevalence of stunting was 39.8%. Stunting increased 2.16 times risk of developing IDA (95% CI = 1.05–4.46).
Cobayashi et al. (2013) [15]	Cross-sectional study	Brazil	1139	Children aged < 10 years old	Serum ferritin < 12 μg/L with hemoglobin < 110 g/L in children aged < 5 years or serum ferritin < 15 μg/L with hemoglobin < 115 g/L in children aged ≥ 5 years old	WHO Growth Standards HAZ < −2.0 SD	10.3% of the subjects had IDA. The prevalence of stunting was 7.1% in children aged < 5 years and 3.7% in children aged ≥ 5 years old. IDA was not significantly associated with stunting in children aged < 5 years old (PR 1.70 [95% CI 0.82–3.73]; *p* = 0.14).
Chen et al. (2020) [16]	Cross-sectional study	Taiwan	589	Infants aged 1–12 months old	Serum ferritin < 15 ng/mL with hemoglobin < 10.5 g/dL	Not mentioned	3.6% of the subjects had IDA. The body length percentile of the subjects did not significantly differ between normal, ID, and IDA infants (52.9 vs. 55.8 vs. 37.9; *p* = 0.118).
Fancony et al. (2020) [17]	Cross-sectional study	Angola	948	Children aged 6–36 months old	Serum ferritin < 12 μg/L with hemoglobin < 11.0 g/dL if there is no inflammation or serum ferritin < 30 μg/L if there is inflammation (CRP > 5 mg/L)	WHO Growth Standards HAZ < −2.0 SD	19.4% of the subjects had IDA. The prevalence of stunting was 26.7%. Children aged 24–36 months old with moderate-to-severe stunting had 2.6 times more risk of developing IDA (95% CI = 1.09–6.20; *p* = 0.031).
Flora et al. (2022) [13]	Cross-sectional study	Indonesia	170	Children aged 9–12 years old	Not mentioned	Not mentioned	The percentage of children with IDA who were stunted was significantly higher than children without IDA (82.5% vs. 17.5%; *p* < 0.001). Children with stunting had 6.785 times risk of developing IDA.
Al Ghwass et al. (2015) [18]	Cross-sectional study	Egypt	345	Children aged 6 months to 12 years old	Low hemoglobin for age with serum ferritin < 12 μg/L or transferrin saturation < 16%	National Center for Health Statistics reference (NCHS) HAZ < −2 SD	55.6% of the subjects had IDA. The prevalence of stunting was 24%. 24.5% of the children with IDA were stunted. There was no significant difference in stunting status between children with IDA and children without IDA (24.5% vs. 18.5%; *p* = 0.234).
Gwetu et al. (2016) [19]	Cross-sectional study	South Africa	184	Children aged 6–8 years old	Body iron stores < 0 mg/kg with hemoglobin < 11.5 g/dL	WHO Growth Standards HAZ < −2.0 SD	3.8% of the subjects had IDA. The prevalence of stunting was 8.3% in boys and 6.6% in girls. Children with IDA had lower HAZ mean compared to children without IDA, despite not being statistically significant (−1.57 vs. −1.15; *p* > 0.05).
Habib et al. (2016) [20]	Cross-sectional study	Pakistan	7138	Children aged 6–59 months old	Serum ferritin < 12 μg/L with hemoglobin < 110 g/L	WHO Growth Standards HAZ < −2.0 SD	33.2% of the subjects had IDA. The prevalence of stunting was 44.5%. Stunting increased risk of developing IDA by 1.42 times (95% CI = 1.23–1.63; *p* < 0.001).
Hossain et al. (2023) [21]	Cross-sectional study	Bangladesh	372	Children aged 8–9 years old	Not mentioned	Not mentioned	52.7% of the subjects had IDA. Subjects with IDA did not have significantly different HAZ score compared to subjects without IDA (−1.4 vs. −1.41; *p* = 0.75).
Mohamed et al. (2021) [22]	Cross-sectional study	Egypt	80	Children aged 24–71 months old	Serum ferritin < 12 mg/dL with hemoglobin < 11 g/dL	CDC sex-matched charts	Children with IDA had significantly lower height percentile compared to children without IDA (19.15 vs. 33.25; *p* = 0.005).
Msaki et al. (2022) [23]	Cross-sectional study	Tanzania	8014	Children aged 6–59 months old	Hemoglobin < 11.0 g/dL	WHO Growth Standards HAZ < −2.0 SD	58.8% of the subjects had IDA. The prevalence of stunting was 36.6%. Children aged 6–59 months old with stunting had 1.31 times higher risk of developing IDA (95% CI = 1.14–1.5; *p* < 0.001).
Orsango et al. (2020) [24]	Cross-sectional study	Ethiopia	331	Children aged 2–5 years old	Serum ferritin < 12 μg/L with hemoglobin < 11.0 g/dL if there is no inflammation or serum ferritin < 30 μg/L if there is inflammation (CRP > 5 mg/L)	WHO Growth Standards HAZ < −2.0 SD	25% of the subjects had IDA. The prevalence of stunting was 37%. The higher the HAZ score, the lower IDA prevalence (adjusted odds ratio 0.74; 95% CI 0.56−0.98).
Sanlidag et al. (2016) [25]	Retrospective study	Cyprus	89	Infant aged 10–18 months old	Serum ferritin < 15 μg/L with hemoglobin < 10.5 g/dL and Mentzer index > 13	Not mentioned	11.2% of the subjects had IDA. Infants with IDA did not have significant difference in height at one year compared to infants without IDA (78.9 cm vs. 75.2 cm; *p* = 0.11)
Tofail et al. (2013) [26]	Cross-sectional study	Bangladesh	434	Children aged 6–24 months old	Serum ferritin < 12 μg/L with hemoglobin < 110 g/L	WHO Growth Standards HAZ < −2.0 SD	51.8% of the subjects had IDA. Children with IDA were more stunted compared to children without IDA (*p* = 0.001). The HAZ score was significantly lower in children with IDA compared to children without IDA (−2.1 vs. −1.7; *p* = 0.002)
Wirth et al. (2021) [27]	Cross-sectional study	Somalia	1456	Children aged 6–59 months old	Serum ferritin < 12 μg/L with hemoglobin < 110 g/L	WHO Growth Standards HAZ < −2.0 SD	28.6% of the subjects had IDA. Stunted children had 40% more risk of being iron--deficient. About 16% of iron deficiency was related to stunting.

CDC = Center for Disease Prevention and Control; CI = Confidence Interval; CRP = C-reactive protein; HAZ = height for age Z-scores; ID = iron deficiency; IDA = iron deficiency anemia; WHO = World Health Organization.

**Table 2:** Results of grading of recommendations, assessment, development and evaluation (GRADE).

Number of Studies	Certainty Assessment						Number of Patients		Effect		Certainty	Importance
	Study Design	Risk of Bias	Inconsistency	Indirectness	Imprecision	Other Consideration	IDA with Stunting	IDA Without Stunting	Relative (95% CI)	Absolute (95% CI)		
Stunting 15	Cross-sectional Retrospective	Serious	Not serious	Not serious	Not serious	None	1238/2650 (46.7%)	1930/4958 (38.9%)	2.27 (1.30–3.95)	202 more per 1000	⨁⨁◯◯ Low	IMPORTANT

CI = Confidence Interval; IDA = iron deficiency anemia. ⨁ defines the certainty level.

## Data Availability

All data are included in the manuscript.
